# Trends in the Prescribing of Thiazides for Hypertension

**DOI:** 10.1371/journal.pmed.0020113

**Published:** 2005-04-26

Hypertension is common in affluent societies and a major risk factor for heart disease. In Canada, hypertension is the leading primary diagnosis for patient visits to physicians' offices. Beyond recommending lifestyle changes such as losing weight, quitting smoking, and lowering salt and alcohol intake, prescription drugs are indicated in many patients. As a consequence, antihypertensive drugs are the leading category of prescription drugs in Canada, accounting for 20% of prescription drug sales.

Several classes of drugs are available for treatment, including diuretics, ACE inhibitors, and calcium channel blockers. First-line treatment with thiazide diuretics—the oldest and by far the cheapest drug class—has been shown in randomized trials to reduce serious cardiovascular morbidity and mortality with benefits at least as great as first-line treatment with other drug classes.[Fig pmed-0020113-g001]

**Figure pmed-0020113-g001:**
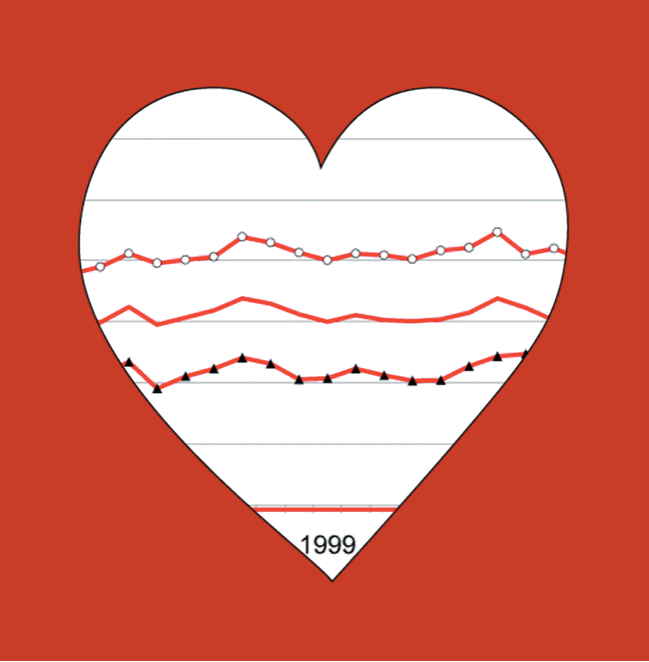
Prescription trends for hypertension

Steve Morgan and colleagues set out to examine whether prescribing practices were in accordance with this evidence. They analyzed administrative claims data from a public drug plan for seniors (residents of age 65 and older) to determine trends in first-line hypertension drug use. During the period from 1993 to 2000, over 82,000 seniors were identified as new users of hypertension drugs. Less than a third of these patients received thiazides (alone or as part of a combination regimen) as a first-line treatment.

The share of new patients receiving a thiazide increased over the study period, but did not exceed 45% at any point. Women were more likely than men, and older patients were more likely than younger ones, to receive thiazides. Comorbidities also influenced prescribing practices: patients without concurrent diagnoses were more likely to receive thiazides. While for some comorbidities (such as previous acute myocardial infarction) evidence suggested that there were good reasons to prescribe drugs other than thiazides, no such evidence existed for many of the other conditions that nevertheless were associated with lower prescription of thiazides.

Changes in drug availability and existing evidence during the period studied make it difficult to calculate the exact extent to which thiazides were under-prescribed. However, the study shows that many patients received drugs that had previously been found to be no better at treating hypertension than much cheaper alternatives. Drug prices changed over the study period as well, but even comparing the lowest price for any of the alternatives to thiazides, $0.34 per day, with the constant cost of less than $ 0.01 per day for a thiazide makes it clear that a lot of money was wasted.

On a more positive note, prescription of thiazides as a first-line therapy rose over the study period—from 25% to 42% in patients without comorbidities. Most of the increase occurred shortly after a specific local education campaign. This suggests that repeated targeting of prescribing physicians—many of whom receive regular marketing material from pharmaceutical companies and subscribe to the general view that newer drugs are better—with current evidence-based information should be considered.

One of the most influential studies comparing antihypertensive drugs, the ALLHAT study, also supported the use of thiazides as first-line drugs. ALLHAT was published in 2003, and its results widely publicized. According to Steve Morgan, “Anecdotal evidence suggests that ALLHAT has had an influence on prescription practices, but I am not aware of a large-scale analysis yet.”

